# Biophysically motivated efficient estimation of the spatially isotropic $R_{2}^{*}$ component from a single gradient‐recalled echo measurement

**DOI:** 10.1002/mrm.27863

**Published:** 2019-07-10

**Authors:** Sebastian Papazoglou, Tobias Streubel, Mohammad Ashtarayeh, Kerrin J. Pine, Luke J. Edwards, Malte Brammerloh, Evgeniya Kirilina, Markus Morawski, Carsten Jäger, Stefan Geyer, Martina F. Callaghan, Nikolaus Weiskopf, Siawoosh Mohammadi

**Affiliations:** ^1^ Department of Systems Neurosciences University Medical Center Hamburg‐Eppendorf Hamburg Germany; ^2^ Department of Neurophysics Max Planck Institute for Human Cognitive and Brain Sciences Leipzig Germany; ^3^ Wellcome Centre for Human Neuroimaging UCL Institute of Neurology London United Kingdom; ^4^ Neurocomputation and Neuroimaging Unit, Department of Education and Psychology Freie Universität Berlin Berlin Germany; ^5^ Paul Flechsig Institute of Brain Research University of Leipzig Leipzig Germany

**Keywords:** anisotropy, apparent transverse relaxation rate, biophysical signal model, gradient‐recalled echo, $R_{2}^{*}$, orientation dependence, white matter

## Abstract

**Purpose:**

To propose and validate an efficient method, based on a biophysically motivated signal model, for removing the orientation‐dependent part of $R_{2}^{*}$ using a single gradient‐recalled echo (GRE) measurement.

**Methods:**

The proposed method utilized a temporal second‐order approximation of the hollow‐cylinder‐fiber model, in which the parameter describing the linear signal decay corresponded to the orientation‐independent part of $R_{2}^{*}$. The estimated parameters were compared to the classical, mono‐exponential decay model for $R_{2}^{*}$ in a sample of an ex vivo human optic chiasm (OC). The OC was measured at 16 distinct orientations relative to the external magnetic field using GRE at 7T. To show that the proposed signal model can remove the orientation dependence of $R_{2}^{*}$, it was compared to the established phenomenological method for separating $R_{2}^{*}$ into orientation‐dependent and ‐independent parts.

**Results:**

Using the phenomenological method on the classical signal model, the well‐known separation of $R_{2}^{*}$ into orientation‐dependent and ‐independent parts was verified. For the proposed model, no significant orientation dependence in the linear signal decay parameter was observed.

**Conclusions:**

Since the proposed second‐order model features orientation‐dependent and ‐independent components at distinct temporal orders, it can be used to remove the orientation dependence of $R_{2}^{*}$ using only a single GRE measurement.

## 1 INTRODUCTION

Quantitative MRI (qMRI) measures in the human brain are typically sensitive to multiple microstructural features at once, e.g., myelin and iron content.^1^, ^2^ The combination of complementary qMRI measures with biophysical models can help to disentangle these different contributions and hence to increase the specificity of qMRI with respect to distinct microstructural properties.^3^ GRE‐MRI is particularly interesting for qMRI since both its magnitude, from which the apparent transverse relaxation rate $R_{2}^{*}$ may be estimated, and its phase, which represents the basis of quantitative susceptibility mapping,^4^, ^5^, ^6^ are sensitive to microstructure. In particular, GRE‐based $R_{2}^{*}$ is sensitive to iron composition, as well as to different axonal properties such as myelination,^7^ and orientation of axons relative to the main field of the MR scanner.^8^, ^9^, ^10^, ^11^, ^12^ The latter could be used to map local fiber direction using GRE measurements at multiple orientations θ of a sample,^13^ but in the case of a GRE measurement acquired at a single head orientation the orientation dependence represents a potential confounder, since the observed $R_{2}^{*}$ would depend on the subject's positioning inside the MR scanner. Hence for GRE‐based $R_{2}^{*}$ to be a robust qMRI parameter this effect needs to be controlled for.

The orientation dependence of GRE‐based $R_{2}^{*}$ can be quantified phenomenologically by partitioning $R_{2}^{*}$ into an orientation‐independent, isotropic component $R_{2,\;\text{iso}}^{*}$ and an orientation‐dependent, anisotropic component $R_{2,\;\text{aniso}}^{*}\left(\theta \right)$, describing the combined effect of bulk susceptibility and microstructure on $R_{2}^{*}$.^14^, ^15^ This could be further explained from biophysical principles by the hollow‐cylinder‐fiber‐model (HCFM).^10^ In the HCFM the observed GRE MR signal is predicted to be the sum of individual signal contributions from the myelin, axonal, and extracellular compartments, and the susceptibility of the myelin sheaths of the white matter (WM) axons is locally described by an anisotropic susceptibility tensor.^10^ To achieve a separation of $R_{2}^{*}$ into isotropic and anisotropic components, either a large number of different orientations of the brain with respect to the external magnetic field have to be acquired,^13^, ^14^, ^16^ or a small number of different orientations of the brain are combined with additional diffusion MRI measurements used to estimate local axonal orientations.^17^ However, both approaches are time consuming and may be difficult to realize in practice.

Inspired by the HCFM, we propose here an efficient method for removing the orientation‐dependent part from $R_{2}^{*}$. Unlike previous methods, our approach requires only GRE data acquired at a single, unknown orientation of the sample. The method is validated in a human postmortem sample of the optic chiasm (OC). Uncomplicated dissection from the postmortem brain and a straightforward anatomy of aligned retinal ganglion cell fibers inside the optic tracts (OTs) make the OC an ideal candidate for this purpose.

## 2 THEORY

Classically,^18^ the GRE signal decay with echo time *TE* is assumed to follow a mono‐exponential function, i.e. the logarithm of the signal may be written as

(1) $$\ln S(TE)=\ln S(0)-\alpha_{1}TE,$$

where $\alpha_{1}\;=\;R_{2}^{*}$ denotes the apparent transverse relaxation rate, *TE* is the echo time and *S*(0) is the signal at *TE* = 0, given by the net magnetization and sensitivity of the MR system. The classical approach to estimating the apparent transverse relaxation rate $R_{2}^{*}$ uses $\alpha_{1}$ in Equation 1 and leads in general to an orientation dependence in $\alpha_{1}\;=\;R_{2}^{*}\left(\theta \right)$, where θ is the angle between local fiber orientation and direction of the external field of the MR scanner.^8^, ^11^, ^17^ To separate $R_{2}^{*}\left(\theta \right)$ into orientation‐independent $\left(R_{2,\;\text{iso}}^{*}\right)$ and orientation‐dependent $\left(R_{2,\;\text{aniso}}^{*}\left(\theta \right)\right)$ relaxation rates, the well‐known phenomenological model can be used^10^, ^11^, ^13^, ^14^

(2) $$R_{2}^{*}\left(\theta \right)=R_{2,\;\text{iso}}^{*}+R_{2,\;\text{aniso}}^{*}\left(\theta \right).$$

where $R_{2,\;\text{aniso}}^{*}\left(\theta \right)\propto \sin^{4}\theta$ for the HCFM.^13^ The isotropic and anisotropic signal contributions could be disentangled on the basis of Equation 2, if GRE‐MRI measurements at multiple, distinct orientations θ of the sample are available.

However, Equation 1 can also be viewed as the first‐order approximation (in *TE*) of the polynomial expansion of the logarithm of a more complex signal expression.

We derived a quadratic signal model inspired by the second‐order expansion of the signal predicted by the HCFM of parallel, hollow cylinders.^13^ Underlying the HCFM is a tissue model for white matter that features three compartments, i.e. extracellular, myelin, and axonal compartments (for a more detailed explanation see the Supporting Information). The signal originating from the myelin is neglected because of its very short $T_{2}^{*}$. The HCFM signal is then assumed to be the sum of the remaining two compartments. Moreover, it is assumed that the static dephasing regime applies. Under these assumptions the HCFM signal simplifies to

(3) $$\ln S(TE)=\ln S(0)-\beta_{1}TE-\beta_{2}TE^{2},$$

where, according to the predictions of the HCFM, $\beta_{1}$ is orientation‐independent, whereas $\beta_{2}$ is orientation‐dependent, following a $\sin^{4}\theta$ function (for more details we refer to our Supporting Information Figures S1 and S2 and our Supporting Information text or to the original HCFM paper.^13^ Consequently, the second‐order model Equation 3 readily leads to a complete separation of orientation‐dependent and ‐independent signal contributions based only on a *single* GRE measurement of $R_{2}^{*}$. Furthermore, it is consequently reasonable to assume that $\beta_{1}\;=\;R_{2,\;\text{iso}}^{*}$, whereas $\beta_{2}$ is exclusively related to $R_{2,\;\text{aniso}}^{*}(\theta )$. To test our hypothesis, we therefore estimated not only the isotropic and anisotropic components in the first‐order coefficients ($\alpha_{1},\beta_{1}$), but also analyzed the second‐order coefficient $\beta_{2}$ using a general orientation dependence in Equation 2

(4) $$k_{j}=k_{j,\;\text{iso}}+k_{j,\;\text{aniso}}\sin^{4}\theta ,$$

allowing for isotropic ($k_{j,\;\text{iso}}$) and anisotropic contributions ($k_{j,\;\text{aniso}}$) to both first‐ and second‐order coefficients $k_{j}\;=\;\alpha_{1},\;\beta_{1},\;\beta_{2}$, in analogy to previous studies.^13^, ^14^, ^16^, ^17^, ^19^

## 3 METHODS

### 3.1 Optic chiasm preparation

**Figure 1:**
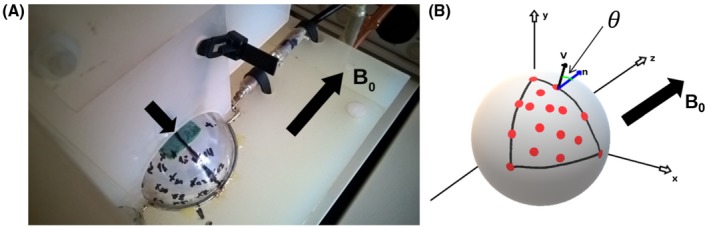
OC sample inside the acrylic sphere with markings for 16 orientations. The arrow above the sphere points at the chosen orientation, while the second arrow indicates the direction of the main field of the MR scanner. B, Schematic of the acrylic sphere in A, showing the assembly of markings arranged on a spherical triangle. The angle θ is defined by the direction of the MR scanner main field **n** and the vector $v_{l}$, which points along the long principal axis of ROI in the right arm of the optic chiasm shown in Figure 2

For validating the proposed method in ex vivo white matter tissue, the orientation dependence of the GRE signal was assessed in an OC sample from a patient without diagnosis of neurological disease (male, 59 year, multi‐organ failure, 48 hours postmortem interval). The OC represents an ideal sample since the axons inside the optic tract (OT) form bundles of parallel fibers with a well‐defined direction θ relative to the main magnetic field. The OC was dissected from a brain provided by the body donation program (see Acknowledgments). The entire procedure of case recruitment, acquisition of the patient's personal data, the protocols and the informed consent forms, performing the autopsy, and handling the autopsy material have been approved by the responsible authorities (Approval by the Sächsisches Bestattungsgesetz von 1994, 3. Abschnitt, §18, Ziffer 8; GZ 01GI9999‐01GI0299; Approval # WF‐74/16, Approval # 82‐02 and Approval # 205/17‐ek). Following the standard Brain Bank procedures, the optic chiasm was immersion‐fixed in (3% paraformaldehyde +1% glutaraldehyde) in phosphate‐buffered saline (PBS) pH 7.4 with three changes of fixation solution on days 7, 14, and 21. In total, the OC remained in the fixation solution for 78 days. The OC was placed inside an acrylic sphere of 60 mm diameter filled with agarose (1.5% Biozym Plaque Agarose (low melting) in PBS + 0.1% sodium). The sphere was manually prepared with markings for 16 different orientations inside a spherical triangle as shown in Figure 1A). One marking corresponded to the case in which fibers inside the right OT of the OC sample and main magnetic field were approximately aligned defining the orientation $\theta_{0}\;=\;0$. However, it was assumed that the fibers in the left OT were also aligned implying that there is no need to model an additional phase offset in Equation 4. This assumption was verified by statistical analysis explained later in the corresponding subsection. The remaining markings were distributed across the spherical surface segment shown schematically in Figure 1B), so that a polar and azimuthal range of *π*/2 was covered. The sphere was manually oriented according to the markings. The OC was scanned at each orientation using the gradient echo sequence described in the next subsection.

### 3.2 Magnetic resonance imaging

All MRI was performed at the Max Planck Institute for Human Cognitive and Brain Sciences on a 7T Siemens Magnetom MRI scanner (Siemens Healthcare GmbH, Erlangen, Germany) using a custom 2‐channel transmit/receive mini‐CP coil with a diameter of 60 mm. GRE signal decay was measured using a 3D gradient echo sequence with *M* = 16 echoes at equally spaced echo times *TE* = 3.4 to 53.5 ms (step size 3.34 ms). Further imaging parameters were: repetition time *TR* = 100 ms, *FoV* = 39 × 39 × 39 mm, matrix size 112 × 112 × 112, flip‐angle 23${\;}^{\circ }$ and a bandwidth of 343 $s^{-1}$/px resulting in 20:59 min total acquisition time for a single orientation of the sample.

### 3.3 Fiber orientation mapping

For estimating the angle between fibers and main magnetic field for each of the of 16 orientations, the gradient echo images corresponding to the first echo time were manually coregistered to the $\theta_{0}\;=\;0$ image using the 3D slicer software.^20^ The corresponding transformation matrices were stored in the header and used for computing the angles according to

(5) $$\theta_{l}=\arctan\left(n\cdot v_{l}\right),$$

(see also Figure 1B). The corresponding values of $\theta_{l}$ were (for *l* = 0, 1, …, 15 and in ${\;}^{\circ }$): 0, 38.7, 49.4, 53.7, 57.3, 59.1, 61.5, 72.6, 83.1, 83.6, 87.7, 88.3, 89.4, and 89.5. In the following the subscript l is suppressed, i.e. θ is used throughout.

### 3.4 Parameter estimation

**Figure 2:**
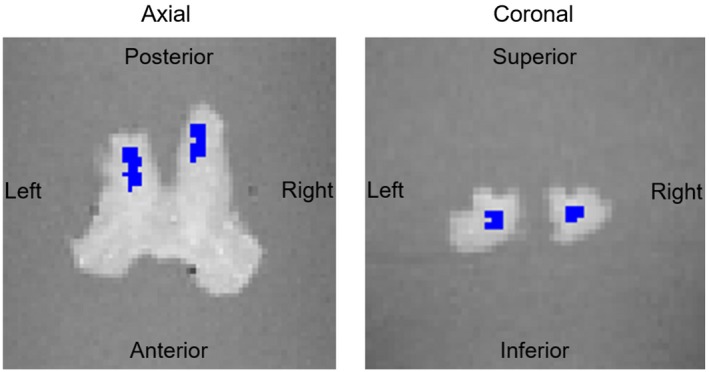
The delineated ROIs shown in an axial and coronal slice of the OT part of the OC sample

For analyses two regions‐of‐interest (ROI) were manually segmented in the left and right OT as shown in Figure 2. Voxel count was 77 and 73 inside the left and right ROI, respectively. The parameters $\alpha_{1}$ from the classical model Equation 1 and $\beta_{1},\;\beta_{2}$ from the proposed second‐order model Equation 3 were estimated in the OC for each θ using a customized version of the hMRI toolbox^21^ and SPM12 together with Matlab R2017a (The MathWorks Inc., Massachusetts).

### 3.5 Analyses

First, the left and right ROI were tested for any significant differences using a *t*‐test on the residuals of $\alpha_{1}$ from Equation 4, since misalignment between fibers in the two ROIs would lead to significant difference in the mean of the residuals of $\alpha_{1}$ from Equation 4. Then, two different analyses were performed to assess the orientation dependence of the model parameters $\alpha_{1},\;\beta_{1}$, and $\beta_{2}$. In the first analysis, the relative contributions of the isotropic and anisotropic components of the model parameters were compared. For this comparison, mean values of the model parameters inside the ROIs were calculated. They were then separated into isotropic ($k_{j,\;\text{iso}}$) and anisotropic ($k_{j,\;\text{aniso}}$) parts ($k_{j}\;=\;\alpha_{1},\;\beta_{1},\;\beta_{2}$) according to the sinusoidal model introduced in Equation 4 using an in‐house Matlab function. From this analysis, the parameters, their corresponding standard deviations (square root of the covariances), and *P*‐values were obtained. In the second analysis, the parameters of both models Equation 1 and Equation 3 were tested for orientation dependence as postulated from Equations 2 and 4. This was achieved by correlating the estimated parameters for both models with $\sin^{4}\theta$ using Matlab's corr function from the statistics toolbox yielding Pearson's *ρ* and a corresponding *P*‐value (*P* < 0.001 was considered significant).

## 4 RESULTS

**Figure 3:**
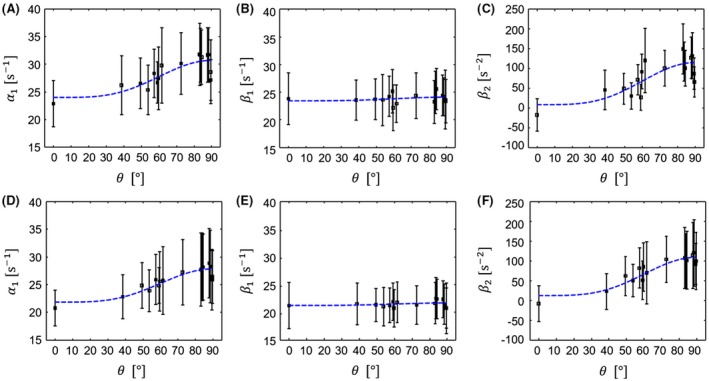
Mean values of the coefficients $\alpha_{1},\;\beta_{1},\;\beta_{2}$ estimated according to the models Equations 1 and 3 inside the ROIs shown in Figure 2 as a function of fiber orientation θ with respect to the magnetic field direction. Errorbars represent standard deviations across voxels in the ROIs. A, B and C correspond to the left ROI, while D, E and F correspond to the right ROI. Results for the linear model (Equation 1) are shown in A and D. The results for the quadratic model (Equation 3) are shown in B, C, E, and F. Dashed blue lines correspond to fits to the data according to Equation 4. The estimated fit parameters are summarized in Table 1

The orientation dependence of the model parameters $\alpha_{1}$, $\beta_{1}$, and $\beta_{2}$ from Equations 1 and 3 is shown in Figure 3 and summarized in Tables 1 and 2. For the linear model Equation 1, the apparent transverse relaxation rate $\alpha_{1}$, which corresponded to the classical $R_{2}^{*}$ clearly showed dependence on θ (Figure 3A and D). In contrast, the first‐order coefficient $\beta_{1}$ of the quadratic model Equation 3 was rather independent of θ (Figure 3B and E), while orientation dependence was clearly observable in the second‐order coefficient $\beta_{2}$ (Figure 3C and F). No significant differences between left and right $\alpha_{1}$‐residuals were found, suggesting that on average fibers in both ROIs were mutually aligned.

**Table 1:** Table summarizing the numerical values of the fit parameters, their standard deviations (in brackets), and the corresponding *P*‐value, from fitting data to Equation 4. These parameters were used to generate the blue lines in Figure 3

		Left ROI	Right ROI
$\alpha_{1}$	$\alpha_{1,\;\text{iso}}$	23.99 (0.88) $s^{-1}$	21.9 (0.53) $s^{-1}$
		*P* < 0.001	*P* < 0.001
	$\alpha_{1,\;\text{aniso}}$	6.76 (1.17) $s^{-1}$	5.99 (0.7) $s^{-1}$
		*P* < 0.001	*P* < 0.001
$\beta_{1}$	$\beta_{1,\;\text{iso}}$	23.50 (0.49) $s^{-1}$	21.21 (0.34) $s^{-1}$
		*P* < 0.001	*P* < 0.001
	$\beta_{1,\;\text{aniso}}$	0.65 (0.66) $s^{-1}$	0.44 (0.45) $s^{-1}$
		*P* = 0.3351	*P* = 0.3474
$\beta_{2}$	$\beta_{2,\;\text{iso}}$	8.62 (16.57) $s^{-2}$	12.06 (7.72) $s^{-2}$
		*P* = 0.6101	*P* = 0.1405
	$\beta_{2,\;\text{aniso}}$	107.31 (22.02) $s^{-2}$	97.62 (10.26) $s^{-2}$
		*P* < 0.001	*P* < 0.001

**Table 2:** Table summarizing the correlations between the coefficients $\alpha_{1},\beta_{1},\beta_{2}$, and $\sin^{4}\theta$ expressed by Pearson's *ρ* and the corresponding *P*‐value

	Left ROI	Right ROI
$\alpha_{1}$	*ρ* = 0.8391	*ρ* = 0.9163
	*P* < 0.001	*P* < 0.001
$\beta_{1}$	*ρ* = 0.2578	*ρ* = 0.2515
	*P* = 0.3351	*P* = 0.3474
$\beta_{2}$	*ρ* = 0.7932	*ρ* = 0.9306
	*P* < 0.001	*P* < 0.001

The results of the first analysis are summarized in Table 1. Going from the linear to the quadratic model, the isotropic part of the first‐order term was essentially unaffected, i.e. $\alpha_{1,\;\text{iso}}\;\approx \;\beta_{1,\;\text{iso}}$ and both parameters were significant. Conversely, its anisotropic part decreased by an order of magnitude: $\alpha_{1,\;\text{aniso}}\;\approx \;10\cdot \beta_{1,\;\text{aniso}}$ and furthermore, $\beta_{1,\;\text{aniso}}$ was not statistically significant. The anisotropic part of the second‐order coefficient $\beta_{2,\;\text{aniso}}$ was much larger and statistically significant in contrast to its isotropic part $\beta_{2,\;\text{iso}}$, supporting our hypothesis that $\beta_{2}$ accounts for effects of anisotropy.

This hypothesis is further supported by the correlation analysis (second analysis) of the coefficients $\alpha_{1},\;\beta_{1},\;\beta_{2}$ with $\sin^{4}\theta$ (see Table 2). It showed that for the quadratic model, the orientation dependence was essentially isolated in the second‐order coefficient $\beta_{2}\;\approx \;\beta_{2,\;\text{aniso}}\sin^{4}\theta$, while the first‐order coefficient $\beta_{1}\;\approx \;\beta_{1,\;\text{iso}}$ was independent of orientation.

## 5 DISCUSSION

In this study, we have introduced a novel method to separate orientation‐dependent and ‐independent contributions to the GRE‐MRI signal decay using only a single GRE experiment. Our method was inspired by the predictions of the biophysical hollow‐cylinder‐fiber model of the GRE signal for short echo times.^10^, ^13^ A Taylor expansion of the GRE signal in the HCFM up to second order in *TE* suggested that orientation dependence should only be observed in the second order, while the linear term is expected to be orientation‐independent. Motivated by this prediction, we employed a second‐order polynomial in *TE* to fit the logarithm of the GRE‐MRI signal, and demonstrated that the coefficient of the first‐order term in *TE* described the orientation‐independent component of the apparent transverse relaxation rate $\left(R_{2,\;\text{iso}}^{*}\right)$, and the second‐order coefficient is related to $R_{2,\;\text{aniso}}^{*}\left(\theta \right)$.

In order to validate the proposed method, we measured GRE‐based $R_{2}^{*}$ at multiple sample orientations in the OTs of the ex vivo human OC sample. Mean values of parameters inside the ROIs (Figure 2) were estimated using the classic mono‐exponential model (Equation 1) and the proposed second‐order model (Equation 3) and tested for potential orientation dependence. The relative magnitude and the statistical significance of the separated parameters according to Equation 4 (Table 1) clearly suggested that the orientation‐independent relaxation is accounted for in the first‐order coefficient $\beta_{1}\;\approx \;\beta_{1,\;\text{iso}}\equiv R_{2,\;\text{iso}}^{*}$. Conversely, the second‐order coefficient accounted for orientation dependency $\beta_{2}\;\approx \;\beta_{2,\;\text{aniso}}\sin^{4}\theta$, and thus was related to $R_{2,\;\text{aniso}}^{*}\left(\theta \right)$. This observation was further supported by a correlation analysis of all coefficients $\alpha_{1},\;\beta_{1},\;\beta_{2}$ to the well‐known $\sin^{4}\theta$‐dependence. Only the first‐order coefficient in the second‐order model, $\beta_{1}$, showed no significant orientation dependence (Table 2). Hence, our method successfully divides orientation‐dependent and ‐independent parts of $R_{2}^{*}$.

Moreover, our results for the magnitude of the anisotropic part of effective transverse relaxation $R_{2,\;\text{aniso}}^{*}\left(\theta \right)$ were in accordance with previous findings in an ex vivo corpus callosum sample at 7T, Lee et al reported 6.4 ± 0.15 $s^{-1}$,^14^ while we found 6.4 ± 0.57 $s^{-1}$ averaged over the left and right ROI (Table 1). In contrast, their values reported for the isotropic component $R_{2,\;\text{iso}}^{*}$ in the corpus callosum were significantly larger (around 50 $s^{-1}$) than observed in our study in the OC (average over the left and right ROI: $23\;\pm \;1.5\;s^{-1}$). One source for increased $R_{2}^{*}$ could be due to the known $R_{2}$ increase in aldehyde fixative solutions due to chemical exchange.^22^ The fact that it can be partly reversed after washing the sample in PBS points toward the fact that this $R_{2}$ increase is related to the interaction between water and formaldehyde monomers.

In,^14^ the tissue was fixed in formalin for approximately one year (concentration was not documented), while in this study the OC was fixed using paraformaldehyde (3%) and glutaraldehyde (1%) and stored in PBS thereafter for 24 days before MR imaging. This interpretation is further supported by the analysis of a second chiasm (data not shown), which was fixed with 4% paraformaldehyde only and in which we observed significantly (*P* < 0.001) larger values only for $R_{2,\;\text{iso}}^{*}$ ($\;\approx \;32\;s^{-1}$).

One limiting factor of this experiment is that the HCFM neglects the direct contribution of the myelin compartment. This is justified by observing that the volume fraction of myelin is much smaller than the volume fractions of axonal and extracellular spaces and, furthermore, the relaxation time of myelin ($T_{2}^{*}\;\approx \;8$ ms) is small compared to echo times typically employed in GRE.^13^ Since the shortest echo time employed in this study (*TE* = 3.4 ms) is well within this range, a contribution of the fast‐relaxing water pools also cannot be excluded. Furthermore, it was demonstrated using simulations that apart from static sources of $R_{2}^{*}$, diffusion‐driven decoherence could lead to a $\sin^{4}\theta$ orientation dependence in $R_{2}$.^23^ This effect might not be observable at room temperature but could become important in in vivo tissue. Moreover, if the upper limit of validity (see α in Equation 9 in the Supporting Information) of the proposed model was estimated using susceptibility values reported in,^10^, ^13^ this would result in α = 36 ms, which is exceeded by the five largest echo times employed in this study. However, if only the shortest eleven echo times with *TE* ≤ 36.8 ms were used, the results reported in Figure 3 and Tables 1 and 2 would remain the same apart from becoming noisier (data not shown), suggesting that the magnetic susceptibilities estimated in^10^ differ from the actual susceptibilities of our OC sample. These differences could be due to differences between in vivo and ex vivo, e.g., due to autolysis or fixation effects, or because they examined the entire white matter, while we examined a small OC specimen. Hence, we cannot rule out in principle an orientation‐dependent contribution to the linear term in the experimentally defined regime. However, our correlation analysis suggested that any potential orientation dependence in the first‐order coefficient of the second‐order model, $\beta_{1}$, (e.g., due to the myelin compartment) was below the sensitivity of this experiment.

Another possible source of orientation dependence in the GRE signal is represented by the bulk magnetic susceptibility of the OC.^4^, ^24^, ^25^ The alteration of $R_{2}^{*}$ is driven by local distortions of the magnetic field, affected by the isotropic and anisotropic bulk susceptibility distribution in the OC, as well as by anisotropic susceptibility resulting from the microscopic structure of the myelin sheaths, and to some extent, by exchange.^15^ Although, it was also shown in,^15^ that the effect of bulk anisotropic susceptibility is four times smaller than the effect of anisotropic microstructure on the frequency shift, its relative contribution on the orientation dependence of the magnitude of the GRE decay is unknown. Finally, also local iron accumulations, in particular in the cortex, could represent another source of a second‐order decay in *TE*.^26^ Clearly, further investigations would be necessary to fully disentangle the signal contributions due to bulk anisotropy, microstrucural anisotropy, diffusion, and iron.

For in vivo application, it should be noted that the proposed method is only formally applicable in white matter consisting of parallel fibers, since the second‐order time dependence in the proposed signal model originates solely from parallel, hollow cylinders. Most voxels in the brain feature more complex fiber configurations than the parallel fibers assumed here, which will have to be accounted for in a revised model, e.g., by including fiber dispersion^15^ or by spherical (de)convolution.^27^ Finally, whether the proposed method can successfully partition $R_{2}^{*}$ into orientation‐independent and dependent parts will depend on both the SNR and the degree of anisotropy of the susceptibility of the tissue.

In the present study, we have introduced and validated an efficient method for removing the orientation‐dependent part of the GRE‐based $R_{2}^{*}$. As compared to previous methods that separated $R_{2}^{*}$ into an orientation‐dependent and ‐independent components,^10^, ^13^, ^14^, ^16^, ^17^ our method can be used without extra measurements to determine fiber orientation and could serve as an efficient way for reducing confounding orientation effects in practice. In particular, it could facilitate studies of the superficial white matter near the cortex using $R_{2}^{*}$, since it will help to reduce the impact of the orientation changes due to gyrifications.^28^ Future studies will have to demonstrate how the method translates to lower field strengths and to in vivo application.

## Supporting information


**FIGURE S1** Definitions of the white matter volume fractions in the HCFM and the *g*‐ratio **FIGURE S2** Schematic of a hollow‐cylinder‐fiber in the static magnetic field of the MR scanner. The vectors **n** and **v** denote the direction of the magnetic field $B_{0}$ of the MR scanner and the principal axis of the fiber, respectively
