# Supplementary material for: Biophysically motivated efficient estimation of the spatially isotropic R2* component from a single gradient‐recalled echo measurement
Source: Magn Reson Med. 2019 Jul 10;82(5):1804–11. doi: 10.1002/mrm.27863 (PMC6771860; doi:10.1002/mrm.27863)
Supplement: Supplementary file 1 — FIGURE S1 Definitions of the white matter volume fractions in the HCFM and the g‐ratio FIGURE S2 Schematic of a hollow‐cylinder‐fiber in the static magnetic field of the MR scanner. The vectors n and v denote the direction of the magnetic field B0 of the MR scanner and the principal axis of the fiber, respectively [file MRM-82-1804-s001.pdf]

Supporting Information

Wharton and Bowtell's microscopic theory uses a hollow cylinder fibre model (HCFM) to describe the gradient-recalled echo (GRE) signal decay [1,2]. In the HCFM, the complex MRI signal consists of three compartments (see Supporting Information Figure S1): $S = S_M + S_A + S_E$, where S_M, S_A, and S_E are the signal contributions from the myelin ($V_M = FVF(1-g^2)$), axonal ($V_A = FVFg^2$), and extra-cellular ($V_E = 1 - FVF$) compartments, respectively, V_x is the WM volume fraction of compartment $x \in \{M, A, E\}$, g is the g-ratio and FVF is the fibre-volume fraction. Assuming the contribution of the myelin water compartment to be negligible, they found the magnitude of the signal to be [2]

$$|S(t)| \approx e^{-R_2^* t} \sqrt{V_A^2 + V_E^2 e^{-2D_E} + 2V_A V_E e^{-D_E} \cos(\omega_A t)}, \quad (1)$$

where R_2^* is the apparent transverse relaxation rate in the absence of the fibres. The average frequency offset induced in the axonal compartment due to the difference of the isotropic and anisotropic magnetic susceptibility of the myelin sheath from the surrounding compartments, ω_A, is given by [1]:

$$\omega_A = \frac{3}{4}\chi_A \sin^2(\theta) \ln\left(\frac{1}{g}\right)\gamma B_0, \quad (2)$$

and the functional form of the effect of dephasing due to the inhomogeneous field in the external compartment, D_E, is assumed to be [3]

$$D_E = \begin{cases} \frac{FVF}{16} |\chi_D|^2 \gamma^2 B_0^2 \sin^4(\theta) t^2 & t \le \alpha \\ \frac{FVF}{2} |\chi_D| \gamma B_0 \sin^2(\theta) \left(t - \frac{2}{|\chi_D|\gamma B_0 \sin^2(\theta)}\right) & t \ge \alpha \end{cases} \quad (3)$$

where θ is the orientation of the hollow cylinder with respect to the external magnetic field B_0 (Supporting Information Figure S2), the time α at which dephasing will not be linear but quadratic in time is

$$\alpha = \frac{3}{|\chi_D| \gamma B_0 \sin^2(\theta)}, \quad (4)$$

and the effective susceptibility χ_D of the hollow cylinder in the external compartment is

$$\chi_D = \left(\chi_I + \frac{\chi_A}{4}\right)\left(1 - g^2\right), \quad (5)$$

with χ_A and χ_I the anisotropic and isotropic susceptibility, respectively. Further details regarding the assumptions of the model may be found in the appendix of [2].

Assuming that t is small enough that D_E is in the quadratic dephasing regime (i.e. $t < 36$ ms at 7T [2]), the second-order Taylor expansion of equation 1 with respect to t is

$$\ln|S(t)| \approx \ln(V_A + V_E) - R_2^* t - \frac{V_A V_E D_E + V_E^2 D_E + 2V_A V_E \omega_A^2 t^2}{(V_A + V_E)^2}. \quad (6)$$

Explicitly, equation 6 can be expressed as

$$\ln|S(t)| \approx \beta_0 - \beta_1 t - \beta_2 t^2, \quad (7)$$

where $\beta_0 = \ln(V_A + V_E)$, $\beta_1 = R_2^*$, and

$$\beta_2 = \frac{V_A V_E d_E + V_E^2 d_E + 2V_A V_E \omega_A^2}{(V_A + V_E)^2} \quad (8)$$

with

$$d_E = \frac{FVF}{16} |\chi_D|^2 \gamma^2 B_0^2 \sin^4(\theta). \quad (9)$$

An expression equivalent in form to equation 6 was derived in [2], where instead of making the small t assumption, a first order expansion in $\omega_A^2 t^2$ and D_E was made under the assumption that these parameters are small in the sense that terms proportional to $(\omega_A^2 t^2)^m (D_E)^n$ could be neglected when $m + n > 1$. We have here opted for the explicit Taylor expansion in small t so that the relation is derived in such a way that no linear or quadratic terms apart from those described above can contribute to the signal decay.

References

[1] Samuel Wharton and Richard Bowtell. Fiber orientation-dependent white matter contrast in gradient echo MRI. *Proceedings of the National Academy of Sciences*, 109(45):18559–18564, November 2012.

[2] Samuel Wharton and Richard Bowtell. Gradient echo based fiber orientation mapping using R2* and frequency difference measurements. *NeuroImage*, 83:1011–1023, December 2013.

[3] Dmitriy A. Yablonskiy and E. Mark Haacke. Theory of NMR signal behavior in magnetically inhomogeneous tissues: The static dephasing regime. *Magnetic Resonance in Medicine*, 32(6):749–763, December 1994.

S1

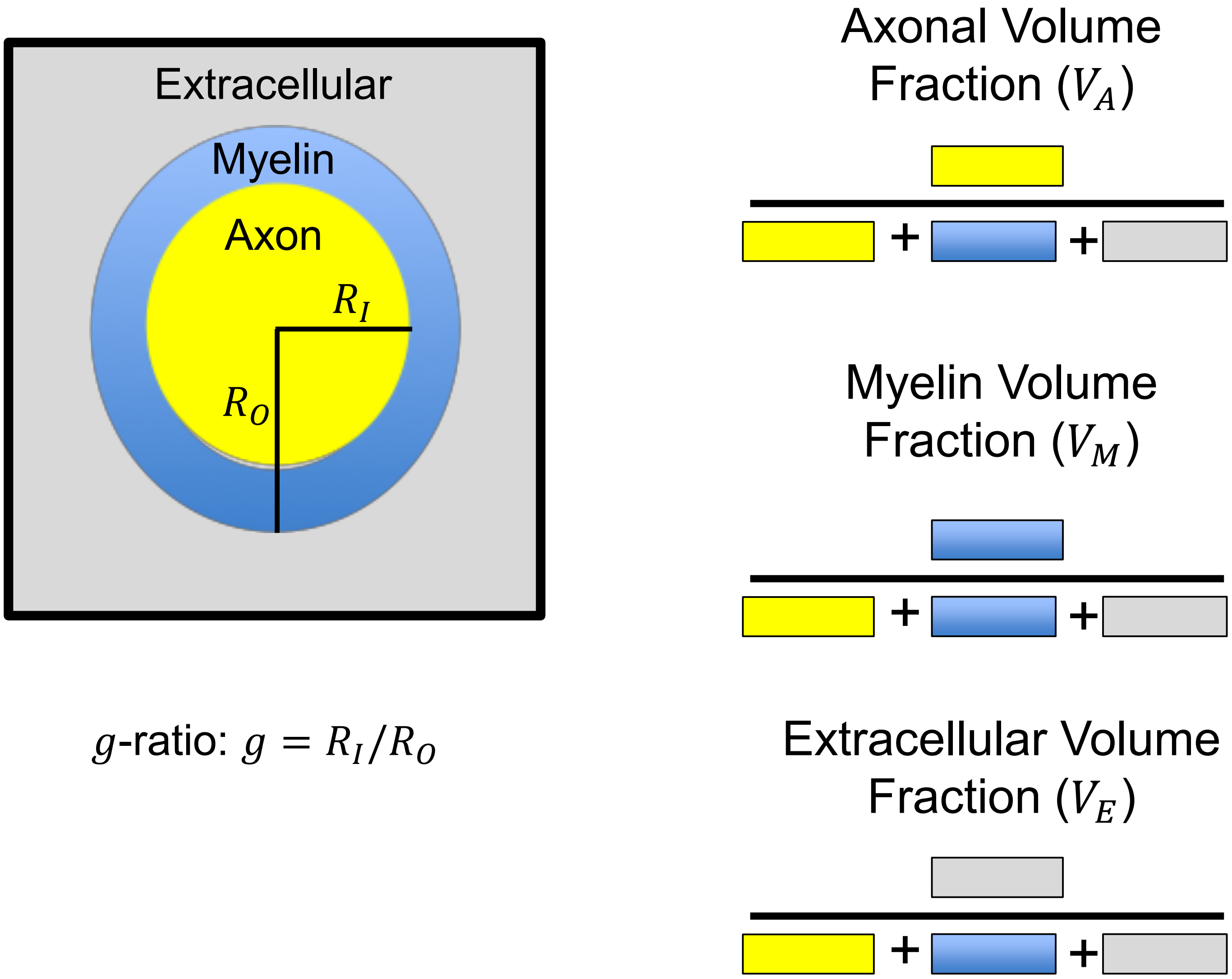
Extracellular
Myelin
Axon
R_I
R_O
g-ratio: $g = R_I/R_O$
Axonal Volume Fraction (V_A)
Myelin Volume Fraction (V_M)
Extracellular Volume Fraction (V_E)

Supporting Information Figure S1: Definitions of the white matter volume fractions in the HCFM and the g-ratio.

S2

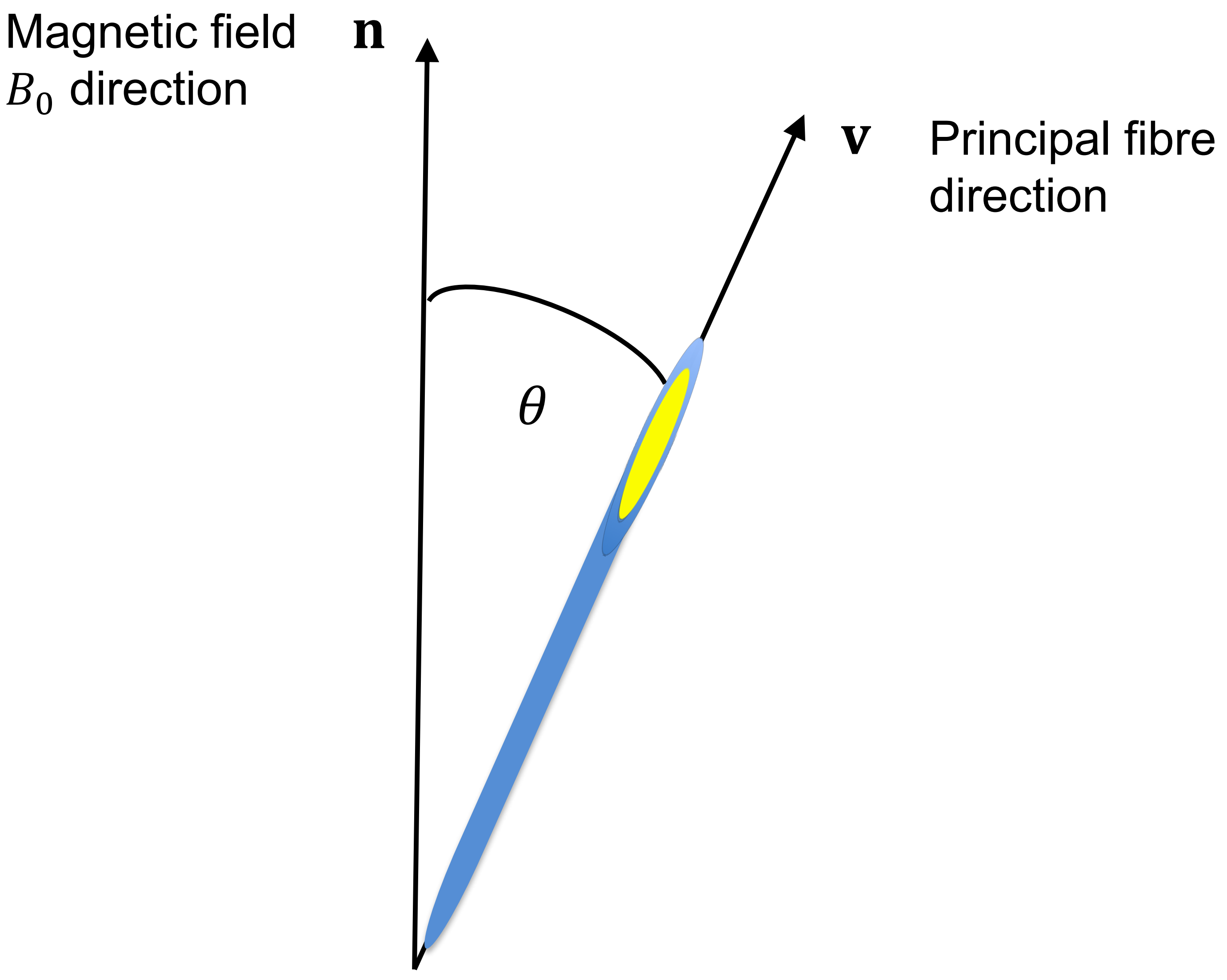

Magnetic field
B_0 direction
$\mathbf{n}$
$\mathbf{v}$
Principal fibre
direction
θ

Supporting Information Figure S2: Schematic of a hollow-cylinder-fibre in the static magnetic field of the MR scanner.
